# *Trem2* Y38C mutation and loss of *Trem2* impairs neuronal synapses in adult mice

**DOI:** 10.1186/s13024-020-00409-0

**Published:** 2020-10-28

**Authors:** Vaishnavi S. Jadhav, Peter B. C. Lin, Taylor Pennington, Gonzalo Viana Di Prisco, Asha Jacob Jannu, Guixiang Xu, Miguel Moutinho, Jie Zhang, Brady K. Atwood, Shweta S. Puntambekar, Stephanie J. Bissel, Adrian L. Oblak, Gary E. Landreth, Bruce T. Lamb

**Affiliations:** 1 Paul and Carole Stark Neurosciences Research Institute, Indiana University, School of Medicine, Indianapolis, IN 46202 USA; 2 Department of Pharmacology and Toxicology, Indiana University, School of Medicine, Indianapolis, IN 46202 USA; 3 Department of Medicine, Indiana University School of Medicine, Indianapolis, IN 462020 USA; 4 Department of Medical and Molecular Genetics, Indiana University, School of Medicine, Indianapolis, IN 46202 USA; 5 Department of Anatomy and Cell Biology, Indiana University, School of Medicine, Indianapolis, IN 46202 USA; 6 Department of Radiology & Imaging Sciences, Indiana University School of Medicine, Indianapolis, IN 46202 USA

**Keywords:** NHD, *Trem2*-Y38C, Early-onset dementia, Transcriptomics, Oligodendrocytes/myelin, Synaptic loss, Synaptic plasticity

## Abstract

**Background:**

Triggering receptor expressed on myeloid cells 2 (TREM2) is expressed in the brain exclusively on microglia and genetic variants are linked to neurodegenerative diseases including Alzheimer’s disease (AD), frontotemporal dementia (FTD) and Nasu Hakola Disease (NHD). The *Trem2* variant R47H, confers substantially elevated risk of developing late onset Alzheimer’s disease, while NHD-linked *Trem2* variants like Y38C, are associated with development of early onset dementia with white matter pathology. However, it is not known how these *Trem2* species, predisposes individuals to presenile dementia.

**Methods:**

To investigate if *Trem2* Y38C or loss of *Trem2* alters neuronal function we generated a novel mouse model to introduce the NHD *Trem2* Y38C variant in murine *Trem2* using CRISPR/Cas9 technology. *Trem2*^Y38C/Y38C^ and *Trem2*^*−/−*^ mice were assessed for *Trem2* expression, differentially expressed genes, synaptic protein levels and synaptic plasticity using biochemical, electrophysiological and transcriptomic approaches.

**Results:**

While mice harboring the *Trem2* Y38C exhibited normal expression levels of TREM2, the pathological outcomes phenocopied *Trem2*^*−/−*^ mice at 6 months. Transcriptomic analysis revealed altered expression of neuronal and oligodendrocytes/myelin genes. We observed regional decreases in synaptic protein levels, with the most affected synapses in the hippocampus. These alterations were associated with reduced synaptic plasticity.

**Conclusion:**

Our findings provide in vivo evidence that *Trem2* Y38C disrupts normal TREM2 functions. *Trem2*^Y38C/Y38C^ and *Trem2*^−/−^ mice demonstrated altered gene expression, changes in microglia morphology, loss of synaptic proteins and reduced hippocampal synaptic plasticity at 6 months in absence of any pathological triggers like amyloid. This suggests TREM2 impacts neuronal functions providing molecular insights on the predisposition of individuals with *TREM2* variants resulting in presenile dementia.

**Supplementary information:**

**Supplementary information** accompanies this paper at 10.1186/s13024-020-00409-0.

## Background

Microglia are brain-resident macrophages that are appreciated for their dynamic nature during inflammation, aging and disease. Recent genome-wide association studies have identified variants of microglial genes that are associated with high risk of developing neurodegenerative diseases, including variants of triggering receptor expressed on myeloid cells 2 (TREM2) [1–4]. TREM2, a single-pass trans-membrane glycoprotein with an extracellular V-type Ig domain and a connecting stalk followed by a transmembrane region and a C-terminal tail. In the brain, TREM2 expression is restricted to microglia [5] and acts to modulate a spectrum of innate immune functions. Upon ligand binding it associates with DNAX-activation protein 12 (DAP12) to initiate downstream signaling [2].

Homozygous loss of function mutations in TREM2 or in its cognate signaling element DAP12, has been reported to cause Nasu Hakola disease (NHD), an autosomal recessive disorder, characterized by early onset dementia and bone cysts [6]. Individuals with the rare R47H variant of *TREM2* have substantial risk of developing late onset Alzheimer’s disease (AD) [7, 8]. This mutation alters the ligand binding region of TREM2, conferring loss of function [9]. Other *TREM2* variants such as Q33X, T66M and Y38C are reported to confer loss of function and have been linked to the development of a frontotemporal dementia (FTD)-like syndrome with early-onset dementia [10]. In vivo, T66M knock-in mice exhibit reduced microglial phagocytosis, cerebral blood flow and brain glucose metabolism [11, 12]. In vitro studies have shown that *Trem2* Y38C mutation have altered protein conformation, which results in retention of TREM2 within the endoplasmic reticulum and less translocation to the plasma membrane [9, 13, 14]. However, it’s in vivo expression pattern remains unknown. A compound heterozygous mutation p.[(Y38C)];[(D86V)] has also been reported in a patient who presented with an FTD-like syndrome [15]. *Trem2* Y38C mutation has also been shown to have altered glycosylation, diminished binding to TREM2 ligands, and impaired phagocytosis in vitro [12, 16–18]. However, the effects of Y38C, in vivo*,* on brain homeostasis in the presence and absence of pathology have yet to be determined.

Beyond the association with neurodegenerative diseases, TREM2 has also been shown to be crucial in brain development. Recent work indicates that *Trem2* deficient mice have increased synapse density at P20 suggesting that TREM2 controls supernumerary synapse pruning by microglia [19]. By 3 months of age, absence of TREM2 leads to altered neuronal connections and an autism spectrum disease-like behavioral phenotype [19]; however, TREM2 mediated mechanisms underlying this neuronal pathology remains unclear. In support of a developmental role for TREM2, the absence of TREM2 in Velocigene *Trem2* knock-out mouse model decreased apposed synapse numbers in the motor cortex at 1 month of age, presumably due to altered communication between astrocytes and *Trem2*-deficient microglia [20]. However, this deficit was normalized by 4 months of age. In the current study, we examine the role of TREM2 during both development and adult stages using two novel mouse models to elucidate how TREM2 influences neuronal functions and can predispose neurons to synaptic alterations. To address this, in the absence of pathological insults like amyloid, we used mice expressing *Trem2* Y38C (*Trem2*^*Y38C/Y38C*^*)* along with those in which *Trem2* gene has been knocked out on a C57BL/6 background (*Trem2*^*−/−*^*)*, allowing determination of whether the Y38C variant exhibited altered function. Using these mouse models, transcriptomics data analysis at 6 months indicated alterations in genes and pathways associated with neuronal functions and oligodendrocyte/myelin, thus presenting mice models that mimic physiological conditions of patients with *TREM2* variants with FTD-like dementia [10]. We observed expression of TREM2 Y38C and *Trem2* deficiency leads to loss of synaptic proteins post developmentally. Vulnerability of pre-synaptic and post-synaptic elements showed region specific differences, with the hippocampus being affected the most. Furthermore, the loss of synaptic protein impacts hippocampal synaptic plasticity indicating effects of *Trem2*^*Y38C/Y38C*^ and *Trem2*^*−/−*^ on neuronal functions. Overall, this study demonstrates loss of functional TREM2 shows age dependent, brain region specific synaptic alterations in absence of pathological triggers like amyloid. These results provide insights on how NHD *Trem2* variant predisposes individual to early onset dementia.

## Methods

### Experimental model

To generate the *Trem2*^*Y38C/Y38C*^ mouse model, CRISPR/Cas9-mediated insertion of the SNP encoding the *Trem2* Y38C variant into the mouse *Trem2* gene was performed as previously reported [21]. Briefly, embryos were injected with Cas9, short-guide RNA (sgRNA) and replacement oligo. The relevant sequences are as follows: *Trem2* targeted region 3′-TACTGCGGAACTTCGTGACCCCC, sgRNA (antisense) 5′- ATGACGCCTTGAAGCACTGGGGG and replacement oligo 3′- TGTGACGCCTTGAAGCACTGGGGA. The first codon in the replacement oligo corresponds to the SNP encoding the Y38C variant, while the third codon corresponds to a silent mutation that ablates the protospacer adjacent motif (PAM) site, necessary for initial binding of CRISPR/Cas9. To identify mice containing the *Trem2* Y38C mutation, Sanger sequencing of founder lines was used to identify mice carrying heterozygous or homozygous *Trem2* Y38C SNP. Five founder lines were selected to generate subsequent crosses. SNP-based genotyping (Thermo Fisher) of offspring was performed using the following primers: forward primer: 5′-GCCGGCCAGTCCTTGAG, reverse primer: 5′-CACCAGGCCTTGCGTCT, SNP reporter 1: 5′-CAAGGCGTCATAAGTACA, SNP reporter 2: 5′-AGGCGTCACAAGTACA. *Trem2* Y38C variant carrying offspring of the founder lines were maintained on a C57BL6/J (B6) background.

*Trem*2^−/−^ (JAX stock #027197) and B6 wildtype (WT) mice were obtained from the Jackson Laboratories (JAX stock #000664). Both male and female mice at ages postnatal day 20 (P20) and 6 months were utilized in this study.

To analyze CRISPR/Cas9 editing efficiency, potential off target mutations were predicted using prediction tool Synthego (https://www.synthego.com/). The top 4 predicted mutations were considered for analysis. Genomic DNA was extracted using 50 mM NaOH from the tail snips of 3 F9 (mice used for the experiments) *Trem2*^*Y38C/Y38C*^ and one WT. Genomic regions with the predicted off-target sites were amplified (Additional file 1: Table S1) and Sanger’s DNA sequencing was performed (GENWIZ) (Additional file 2: Table S2). Sequences were then analyzed for the predicted mutation. No predicted off-target mutations were detected in the *Trem2*^*Y38C/Y38C*^ samples.

Mice were housed in the Indiana University School of Medicine (IUSM) animal facilities, which are accredited by the Association and Accreditation of Laboratory Animal Care. Animals were maintained according to USDA standards and the National Institutes of Health Guide for the Care and Use of Laboratory Animals. Experiments were approved by the IUSM Institutional Animal Care and Use Committee.

### Brain extraction and tissue processing

Mice were deeply anesthetized with ketamine/xylazine and perfused with sterile ice-cold PBS, and their brains were removed. For immunohistochemistry, one hemisphere was drop-fixed in 4% PFA in PBS for 24–48 h, transferred to ice-cold 30% sucrose and stored at 4 °C in in PBS. After embedding in OCT Compound (VWR), 30 μm thick sections were obtained on a Leica CM 1950 cryostat. Cortex and hippocampus was microdissected from the other half of the brain, snap-frozen and stored at − 80 °C until use. Frozen brain tissue (cortex or hippocampus) was homogenized in ice-cold T-PER homogenization buffer (ThermoFisher, 78,510) supplemented with protein phosphatase (1:100, P5726, Sigma-Aldrich) and protease inhibitor cocktail (1:100, P8340, Sigma-Aldrich). Homogenates were aliquoted and reserved for protein extraction or RNA extraction.

### RNA extraction

Homogenized cortical lysates that were prepared in ice-cold T-per homogenization buffer, as described above, were added to an equal volume of QIAzol lysis reagent (QIAGEN RNeasy kits). RNA was isolated using QIAGEN RNeasy kit as per the manufacturer’s protocol and quantified using NanoDrop (Thermo Scientific).

### RNA-seq library preparation

Total RNA from cortices of WT, *Trem2*^Y38C/Y38C^ and *Trem2*^−/−^ mice were first evaluated for its quantity, and quality, using Agilent Bioanalyzer 2100. One hundred nanograms of total RNA were used. Ribosomal RNA was removed from total RNA using QIAseq FastSelect rRNA Removal HMR Kit (Qiagen, Catalog# 334387 Human/Mouse/Rat). After the depletion of rRNA, cDNA library preparation was carried out including RNA fragmentation, cDNA synthesis, ligation of index adaptors, and amplification, following the KAPA RNA Hyper Prep Kit Technical Data Sheet, KR0961 – v3.15 (Roche Corporate, Catalog #KK8541). Each resulting indexed library was quantified and its quality accessed by Qubit and Agilent Bioanalyzer, and multiple libraries were pooled in equal molarity. The library pool was then sequenced in 100b paired-end read format on NovaSeq 6000 (Illumina, Inc.). More than 30 million reads per sample were generated and 91% of the sequencing reads reached Q30 (99.9% base call accuracy). A Phred quality score (Q score) was used to measure the quality of sequencing.

### Mapping QC and data analysis

The sequencing data were first assessed using FastQC (Babraham Bioinformatics, Cambridge, UK) for quality control. All sequenced libraries were mapped to the mouse genome (mm10) (or to the human genome (hg38)) using STAR RNA-seq aligner (v.2.5) [22] with the following parameter: “--outSAMmapqUnique 60”. The reads distribution across the genome was assessed using bamutils (from ngsutils v.0.5.9) [23]. Uniquely mapped sequencing reads were assigned to mm10 refGene genes (or hg38 refGene genes) using featureCounts (from subread v.1.5.1) [24] with the following parameters: “-s 2 –p –Q 10”. Differential expression and visualization were performed using R Bioconductor package DESeq2 by pair-wise comparison of *Trem2*^Y38C/Y38C^ versus WT and *Trem2*^−/−^ versus WT. Counts were normalized and examined for outliers in DESeq2. The list was further filtered based on FDR (P-adj) < 0.05 and Log fold changes (logFC), |logFC| < 0.58. All the filtered differentially expressed genes were annotated using Ingenuity Pathway Analysis (IPA) software. Enrichment for Tissue type, Molecular Functions, Cellular Components, and Pathways on the filtered differentially expressed genes were performed using Enrichr web-server tool [25, 26]. The smallest *P*-value indicates the highest degree of enrichment. Volcano plots and heatmaps were plotted using volcanoPlot and pheatmap functions in R.

### Western blotting

For protein extraction, homogenates of microdissected cortices and hippocampi were sonicated and centrifuged at 50,000×g. Protein concentrations were measured using a Bicinchoninic Acid (BCA) assay (Thermo Fisher, 23,225), according to the manufacturer’s instructions. Proteins were denatured at 95 °C for 10 min in 3X denaturing buffer containing LDS sample buffer and 50 mM DTT. 15–20 μg of protein for each sample were loaded onto Novex 4–12% Bis-Tris gels (Invitrogen) and run at 150 V for 1 h in MES running buffer (Thermo Fischer) and transferred onto PVDF membranes (EMD Millipore) in Tris-Glycine transfer buffer containing methanol at 100 V for 2 h on ice. Membranes were blocked in TBST (Tris-buffered saline with 0.1% Tween 20) containing 5% BSA for 1 h at room temperature and incubated in the indicated primary antibodies in blocking buffer overnight at 4 °C with shaking: PSD-95 (NeuroMab, 75–028, 1:20,000), Synaptophysin (Cell Signaling technology, 5461, 1:20,000), Synapsin 2 (Abcam, ab13258, 1:1000), Homer 1 (GeneTex, 103,278, 1:5000), CNPase (abcam, ab6319,1:500), MBP (abcam, ab7349, 1:2000), β actin (Sigma, A1978, 1:200,000). Membranes were then washed in TBST and incubated for 1 h with appropriate secondary antibody diluted in 5% milk in TBST at room temperature. Membranes were imaged using Chemiluminescence HRP substrate (Millipore). Densitometry was performed using Image J.

### Immunohistochemistry

30 μm free-floating tissue sections were subjected to antigen retrieval in sodium citrate buffer (10 mM, pH 6) for 10 min at 95 °C. After cooling, sections were blocked in blocking buffer (5% Normal Donkey Serum, 0.3% TritonX-100 in PBS) and incubated with the appropriate primary antibody overnight at 4 °C: Iba-1 (Millipore, MABN92 or Wako, 019–19,741​; both at 1:500). After washing, sections were incubated in corresponding Alexa Fluor-conjugated secondary antibodies (1:500). Sections were mounted with ProLong Gold AntiFade with DAPI (Fisher Scientific). For Trem2 immunofluorescent staining, Tyramide Signal Amplification (TSA) Biotin System kit (PerkinElmer, NEL700A001KT) was used. Briefly, following antigen retrieval (as described above), sections were blocked in TNB blocking buffer at room temperature for 1 h. Sections were then incubated in primary antibody for TREM2 (R&D systems, AF1729, 1:300) overnight at 4 °C. After washing in TNT wash buffer, sections were incubated with anti-sheep biotinylated secondary antibody (Vector laboratories, BA-6000, 1:850) mkj for 1 h at room temperature. Sections were washed and incubated in Streptavidin-HRP (1:1000). Sections were washed and then incubated in Tyramide solution (1:250) for 4 min. After washing, the sections were incubated in Streptavidin Alexa Fluor 488 (Invitrogen, S32354, 1:1000) at room temperature for 1 h. After washing, sections were blocked and stained for Iba-1 as described above.

Morphological analysis of Iba-1 positive cells was performed on a total of 4 sections (2 medial tissue sections and 2 lateral sections), from 6 mice (equal number of males and females) per genotype. The number of Iba-1 positive cells and percent area of Iba-1 staining were quantified on 10X images of the entire cortex and hippocampus of a section. After thresholding, soma number and total cell area were quantified in a blinded manner using ImageJ. 31–33 microglia per genotype were selected randomly from the cortical images which were binarized and skeletonized using ImageJ Skeletonize plugin, as described previously [27]. Using Analyze Skeleton feature, total number of branches and junctions per cell were quantified for images from cortex. Immunofluorescent images to visualize TREM2 were acquired with the Nikon AR1 Confocal microscope. Maximum intensity of Z-stacks were obtained using 60X Nyquist view images of microglia from similar cortical regions for all the genotypes to confirm the expression of TREM2 in Iba-1 positive cells.

### Enzyme linked immunosorbent assay (ELISA)

To quantify total TREM2 protein levels, cortices from WT, *Trem2*^*Y38C/Y38C*^ and *Trem2*^*−/−*^ were homogenized in lysis buffer (25 mM Tris pH 7.4, 150 mM NaCl, 1 mM EDTA, 5% glycerol, 1% NP-40) and protein concentration was determined by Bicinchoninic Acid (BCA) assay (Thermo Fisher, 23,225). F8 Maxisorp Nunc-Immuno Module (Thermo Fisher, 468,667) wells were coated with 2 μg/ml of the TREM2 capture antibody (R&D Systems, MAB1729) in 0.05 M carbonate/bicarbonate buffer (pH 9.6), overnight at 4 °C, and blocked with 3% BSA, 0.05% Tween in PBS for 1 h at RT. TREM2 standards were prepared using recombinant mouse TREM2 protein (R&D system, 9228-T2). Standards, and 500 μg of cortical lysates were incubated for 2 h at room temperature. Wells were washed 4 times with 0.05% Tween in PBS and incubated with 0.25μg/ml of the TREM2 biotinylated detection antibody (R&D Systems, BAF1729) for 1 h at RT. After washing, samples were incubated with HRP-conjugated streptavidin (PerkinElmer, NEL750001EA, 1;10,000). The samples were washed and incubated with the chromogenic substrate TMB (3,3′,5,5′-tetramethylbenzidine) (Pierce TMB Substrate kit, ThermoFisher, 34,021). Upon optimal color development, reactions were stopped using 1 N HCL and wells were read at 450 nM using the Epoch2 microplate reader (BioTek). For sTREM2 quantification, frozen mouse cortices were subjected to cryogenic grinding in a mortar and pestle. Cortices were further homogenized in ice-cold Tris-buffered saline (TBS; 50 mM Tris pH 7.4, 150 mM NaCl; 10 w/v) using a 26-G needle and cleared by ultracentrifugation (186,000×g,1 h, 4 °C). Protein concentration was determined using BCA assay and 140 μg of protein was added to the wells. sTREM2 levels were quantified using the ELISA protocol described above.

### Quantitative RT-PCR (qRT-PCR)

cDNA was prepared from 700 ng of RNA using the High Capacity RNA-to-cDNA kit (Applied Biosystems, 4,387,406) and quantitative PCR (qPCR) was performed using the QuantStudio 6 Flex (Applied Biosystems). Murine *Trem2* mRNA quantification was performed using following primers: Forward 5′- CCTCTCCACCAGTTTCTCCT − 3′ and Reverse 5′- CAGTGCTTCAAGGCGTCATAAG − 3′. Taqman assays used: *Mbp* (Mm01266402_m1), *Olig2* (Mm01210556_m1), *Mobp* (Mm02745649_m1). Relative gene expression was determined using the ΔΔCT method and graphed as fold change relative to murine *GAPDH*.

### Brain slice preparations

Mice (6-month-old) were deeply anesthetized using isoflurane and sacrificed by rapid decapitation. Brains were removed and placed in a 95% O_2_ and 5% CO_2_-saturated, ice-cold tissue cutting solution (194 mM sucrose, 30 mM NaCl, 4.5 mM KCl, 1 mM MgCl_2_, 26 mM NaHCO_3_, 1.2 mM NaH_2_PO_4_, 10 mM glucose). Sagittal slices containing hippocampus were cut to a thickness of 350 μm using a vibratome (Leica VT1200S) and transferred to an artificial cerebral spinal fluid (aCSF) (124 mM NaCl, 4.5 mM KCl, 1 mM MgCl_2_, 26 mM NaHCO_3_, 1.2 mM NaH_2_PO_4_, 10 mM glucose, 2 mM CaCl_2_) saturated with 95% O_2_ and 5% CO_2_ at 30 °C for 1 h. Slices were then kept at room temperature until recording.

### Field potential recordings

Field excitatory postsynaptic potential (fEPSP) recordings were performed on brain slices containing the stratum radiatum of hippocampal CA1 region in a chamber that was continuously perfused with aCSF at a rate of 1–2 mL/min in 5% CO_2_ at 30–32 °C. Slices were visualized using an Olympus BX51WI microscope (Olympus Corporation of America). Extracellular recordings were conducted using a Multiclamp 700B amplifier (Molecular Devices). Schaffer collaterals in the hippocampus were stimulated with tungsten stereotrodes (MicroProbes for Life Science). To record responses from hippocampal CA1 neurons, filament-containing borosilicate micropipettes (World Precision Instruments) were prepared using a P-1000 micropipette puller (Sutter Instruments) and filled with 1 M NaCl. A Constant current Isolated Stimulator (Digitimer) was used to produce electrical stimulation.

Input-output (I/O) curves were recorded to determine the maximum stimulus-response, which was determined by calculating the slope of the response (mV/ms). Using the stimulation strength that produced 50% of the maximum intensity, a stable baseline was observed for 10 min before recording the pair pulse ratio (PPR) and field excitatory postsynaptic potential (fEPSPs). The PPRs were obtained every 20 s for a total of 3 min. The fEPSPs were acquired across a 70-min time window: 10 min of pre-stimulation baseline and 60 min post-stimulation. Long term potentiation (LTP) was induced using a high-frequency protocol: 4 trains of 10 pulses, every 20 s at 100 Hz. The investigators were blinded to genotype.

### Statistical analysis

False discovery rates (P-adjusted) and *P* value were calculated using the Benjamini & Hochberg method [28] and Wald test, respectively, within DESeq2. Statistical analyses were performed using Prism (GraphPad) for rest of the data. Statistical significance was determined using a one-way or two-way ANOVA with Bonferroni or Tukey’s post hoc analysis, with *P* values less than 0.05 considered as significant. Fisher’s exact test were used for contingency tables. Each ‘N’ represents a single biological replicate and details for each experiment can be found in the figure legends. Data shown are representative of at least two independent experiments and are represented as the mean and error bars show the standard error of the mean (SEM) unless otherwise noted.

## Results

### *Trem2*^*Y38C/Y38C*^ mouse model generation

**Fig. 1:**
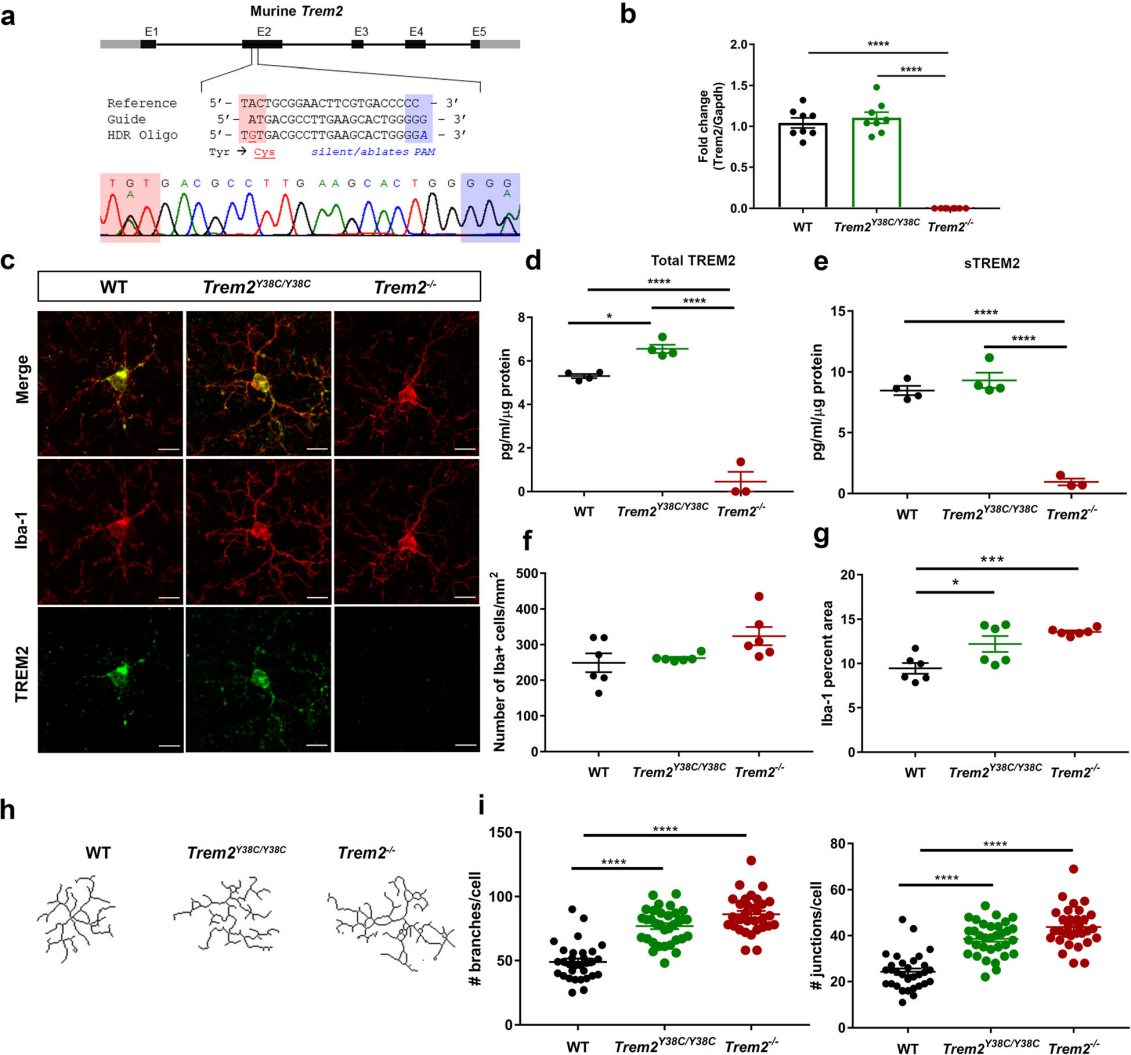
*Trem2* Y38C variant and *Trem2* deficient mice affects microglial morphology. **a** CRISPR/Cas9 strategy to generate the tyrosine to cystine point mutation (red) in exon 2 of murine *Trem2* to create the *Trem2*^*Y38C/Y38C*^ mouse model. The sequences for the reference genome, guide RNA, and homology directed repair (HDR), which indicates the Y38C variant (red) and a silent mutation (blue) to ablate the protospacer adjacent motif (PAM), are shown. **b** Quantification of *Trem2* mRNA levels in cortical lysates showed similar *Trem2* expression in WT (black) and *Trem2*^*Y38C/Y38C*^ (green) mice and no expression in *Trem2*^*−/−*^ (red) mice. Data are presented as mean ± SEM fold change normalized gene expression relative to WT. *N* = 8 (4 males and 4 females for each group) **c** Immunohistochemistry for TREM2 (green) and Iba-1 (red) is shown in cortices of 6 month WT, *Trem2*^*Y38C/Y38C*^, and *Trem2*^*−/−*^ mice. Merged imaged indicates colocalization of TREM2 staining with Iba-1+ (red) cell bodies in WT and *Trem2*^*Y38C/Y38C*^ mice. No TREM2 staining was observed in *Trem2*^*−/−*^ mice. (representative images from females). **d** Quantification of total TREM2 in WT, *Trem2*^*Y38C/Y38C*^, and *Trem2*^*−/−*^ cortical lysates by ELISA indicates slightly higher levels of TREM2 in *Trem2*^*Y38C/Y38C*^ as compared to the WT mice. **e** sTREM2 levels, quantified in TBS soluble cortical extracts, were similar in both *Trem2*^*Y38C/Y38C*^ and WT. **f** Quantification of the number of Iba-1 positive cells showed similar counts in cortices of 6 months old WT, *Trem2*^*Y38C/Y38C*^ and *Trem2*^*−/−*^ mice. **g** Quantification of Iba-1 immunoreactive area showed increased staining in *Trem2*^*Y38C/Y38C*^ and *Trem2*^*−/−*^ mice. **f-g** Data represented as mean ± SEM, *N* = 6 (3 males and 3 females for each group; *n* = 56 fields for WT, *n* = 89 fields for *Trem2*^*Y38C/Y38C*^, and *n* = 112 fields for *Trem2*^*−/−*^). **h** Skeletonized reconstructions (representative reconstructions from females), **i** quantification of number of branches and **j** number of junctions in microglia from the cortices of 6 months old WT, *Trem2*^*Y38C/Y38C*^ and *Trem2*^*−/−*^ mice. **h-j** Data represented as mean ± SEM, N = 6 (3 males and 3 females for each group, each data point represents microglia analyzed). **b,d-i** One-way ANOVA with Tukey’s post hoc test. * *P* < 0.05, ***P < 0.001, ****P < 0.0001

To examine the consequences of a putative loss of function *Trem2* variant, *Trem2*^Y38C/Y38C^ mice containing the NHD *Trem2* Y38C variant were generated using CRISPR/Cas9 to introduce a point mutation in the endogenous *Trem2* gene, resulting in a substitution of tyrosine to cystine at position 38 (Fig. 1a). To investigate the effects of TREM2 deficiency, we used the CRISPR/Cas9 *Trem2*^*−/−*^ mouse model. *Trem2* expression in both models was evaluated using PCR primers flanking the inserted stop codon in *Trem2*^−/−^ mice. *Trem2* mRNA levels were unaffected in *Trem2*^Y38C/Y38C^ mice, while no *Trem2* mRNA expression was observed in *Trem2*^−/−^ mice (Fig. 1b). Since an artifactual alternate splice variant has been reported in the *Trem2* R47H variant mouse model that resulted in reduced *Trem2* mRNA and protein expression [29], we evaluated the presence of alternate splice variants in the *Trem2*^Y38C/Y38C^ mouse model but none were found (data not shown). Additionally, because the Velocigene *Trem2*^−/−^ mouse model shows off-target upregulation of the adjacent *Treml1* gene [30], we examined the expression of genes flanking the *Trem2* locus, 100 kb upstream and downstream, on chromosome 17 in *Trem2*^Y38C/Y38C^ and *Trem2*^−/−^. We did not detect significant aberrant expression of genes within this region (Additional file 3: Table S3). At the protein level, TREM2 was localized to Iba-1+ microglia in the brains of *Trem2*^Y38C/Y38C^ mice and was absent in *Trem2*^−/−^ mice (Fig. 1c). TREM2 protein levels in *Trem2*^Y38C/Y38C^ cortical lysates, when measured by ELISA, were slightly higher than WT and were negligible in *Trem2*^−/−^, indicating that TREM2 protein is expressed and detected in *Trem2*^Y38C/Y38C^ mice (Fig. 1d). sTREM2 levels in TBS soluble cortical lysates of *Trem2*^Y38C/Y38C^ were similar to WT and were scant in *Trem2*^−/−^ mice (Fig. 1e). This suggests, *Trem2*-Y38C mutation did not alter the expression of TREM2 protein in *Trem2*^Y38C/Y38C^ mice.

### *Trem2*^Y38C/Y38C^ and *Trem2*^−/−^ alters microglial morphology

To determine whether the *Trem2-* Y38C variant and deficiency of *Trem2* affects microglia phenotype and number, we examined the total number of Iba-1+ cells and microglial morphology in the cortices of 6 months old WT, *Trem2*^Y38C/Y38C^ and *Trem2*^*−/−*^ mice using immunohistochemistry. The Iba-1+ cell number in both *Trem2*^Y38C/Y38C^ or *Trem2*^*−/−*^ mice were similar to WT (Fig. 1f). *Trem2*^Y38C/Y38C^ and *Trem2*^*−/−*^ microglia both showed increase in the percent area of Iba-1 staining indicating individual cells covered a larger area (Fig. 1g). This could be due to increased microglial branching in *Trem2*^Y38C/Y38C^ and *Trem2*^*−/−*^ mice as represented by the quantification of skeletonized microglia (Fig. 1h-j).

### *Trem2*^Y38C/Y38C^ and *Trem2*^−/−^ mice show alterations in genes related to oligodendrocyte/myelin and neuronal functions

**Fig. 2:**
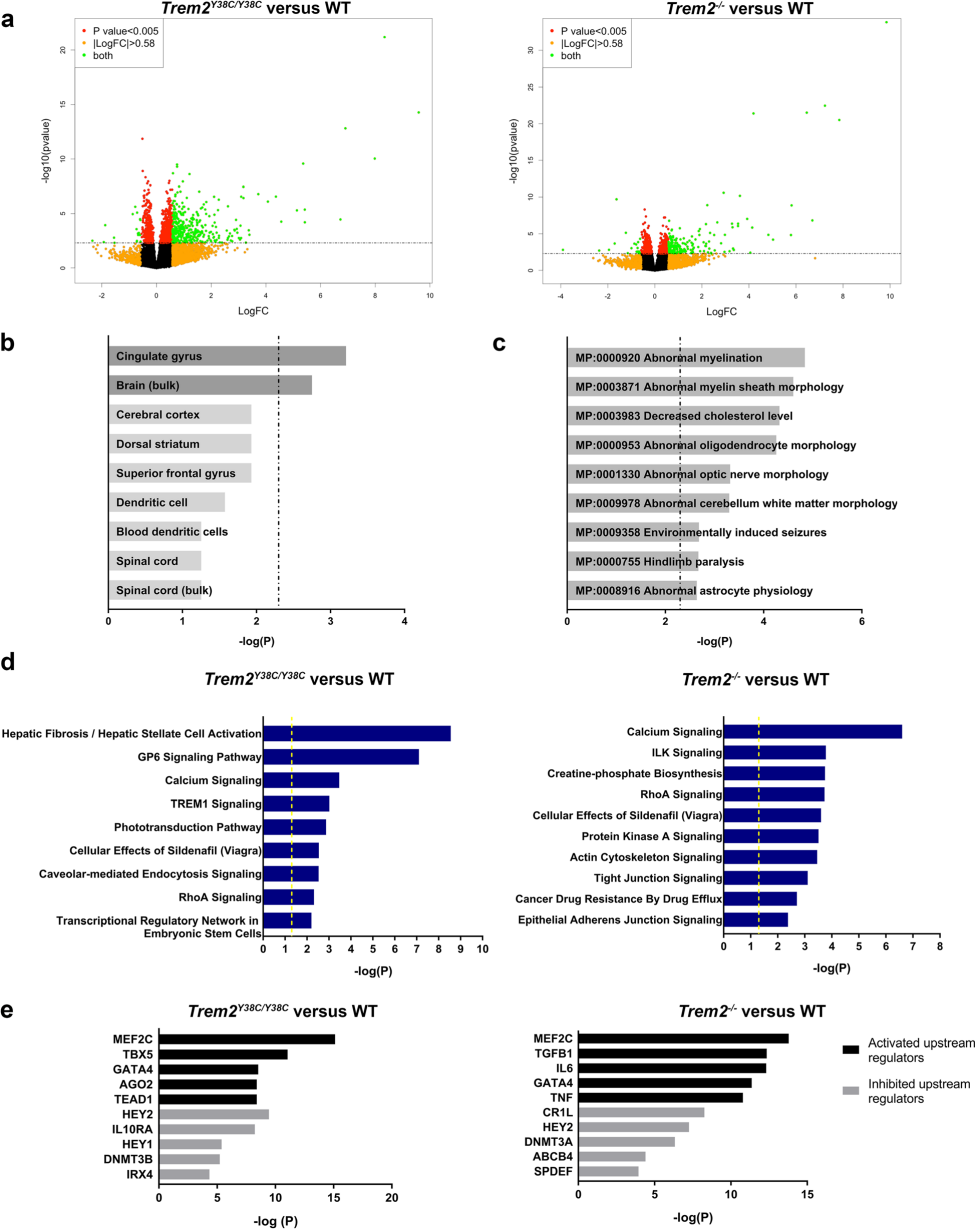
Loss of functional TREM2 alters gene expression associated with oligodendrocyte/myelin and neuronal function. **a** Volcano plot showing differentially expressed genes in 6 month *Trem2*^*Y38C/Y38C*^ versus WT cortices and *Trem2*^*−/−*^ versus WT cortices. Dashed line represents *P* < 0.005. Significantly altered genes are shown in green with downregulated and upregulated genes above |logFC| > 0.58 threshold of in green. **b-c** Enrichr pathway analysis of the shared downregulated genes between *Trem2*^*Y38C/Y38C*^ and *Trem2*^*−/−*^ mice (ranked by *P* value; Fischer’s exact test, P-adjusted, Benjamini-Hochberg method for correction for multiple hypotheses testing). **b** Tissue enrichment analysis showing significant enrichment in brain regions. Dashed line represents *P* < 0.005. **c** Enrichment analysis for specific cellular compartments showing myelin-related enrichment. Dashed line represents *P* < 0.005. **d** Top 10 pathways significantly altered using IPA pathway analysis. Analysis based on significantly differentially expressed genes between *Trem2*^*Y38C/Y38C*^ versus WT mice and *Trem2*^*−/−*^ vs WT mice. Yellow line represents *P* < 0.05 yellow line (ranked by *P* value, Fischer’s extact test). **e** Top 5 predicted upstream regulators of differentially expressed genes in *Trem2*^*Y38C/Y38C*^ versus WT mice and *Trem2*^*−/−*^ vs WT mice using IPA Upstream Regulator analysis. Activated (black) and inhibited (grey) upstream regulators are shown. *P* value, Fisher’s exact test. Sample sizes: WT mice, *N* = 8 (4 males, 4 females); *Trem2*^*Y38C/Y38C*^ mice, *N* = 8 (4 males, 4 females); *Trem2*^*−/−*^ mice, *N* = 5 (1 male, 4 females)

In order to better understand the effects of loss of *Trem2* expression and the *Trem2* Y38C variant on various physiological processes in the brain in an unbiased manner, we performed RNA sequencing to explore differences in gene expression in microdissected cortices of *Trem2*^Y38C/Y38C^, *Trem2*^−/−^, and WT mice at 6 months of age (Fig. 2a, Additional file 4: Figure S1A,B). The list of all differentially expressed genes (DEG) are in Additional files 5 and 6: Table S4 and S5. Compared to WT control mice, we observed 1076 DEG in *Trem2*^Y38C/Y38C^ and 665 DEG in *Trem2*^−/−^. Cortices of *Trem2*^*Y38C/Y38C*^ and *Trem2*^*−/−*^ mice shared, 331 DEG in common (Additional file 4: Figure S1C). We performed gene ontology enrichment analysis on commonly altered genes using Enrichr. Enrichment for cell type indicated the downregulated genes associated with cortical layers 2/3 (Additional file 4: Figure S1D). We found 177 DEGs in *Trem2*^*Y38C/Y38C*^ vs *Trem2*^*−/−*^ cortices (Additional file 7: Table S6, Additional file 8: Figure S2A). To understand the pathways affected, genes (FDR < 0.05) were analyzed for pathway enrichment using Enrichr. Down regulated genes in *Trem2*^*Y38C/Y38C*^ samples enriched in circadian rhythm pathways whereas upregulated genes were enriched in pathways associated with protein processing in endoplasmic reticulum as compared to the *Trem2*^*−/−*^ samples (Additional file 8: Figure S2B-C).

Enrichment analysis of downregulated genes in *Trem2*^Y38C/Y38C^ and *Trem2*^−/−^ samples for cell compartments revealed myelin and axons as top enriched terms as compared to WT samples (Fig. 2c, Additional file 4: Figure S1E). Overall, this analysis highlighted alterations in oligodendrocytes/myelin and neuronal cells. To further characterize the effect of *Trem2*^Y38C/Y38C^ and *Trem2*^−/−^ on oligodendrocyte/myelin gene expression, we analyzed oligodendrocyte/myelin molecular signatures [31–33] to determine what effect *Trem2* variant or loss of function would have on known oligodendrocyte/myelin gene expression patterns. Among these markers, we observed significant reduction in the expression of 17 transcripts in *Trem2*^*Y38C/Y38C*^ and 6 transcripts in *Trem2*^*−/−*^ (Additional file 9.10: Table S7 and S4). White-matter alterations have been reported in NHD patients, including patients with *Trem2-*Y38C homozygous mutations [10].

To explore the pathways that could elucidate the microglial and myelin alterations in *Trem2*^Y38C/Y38C^ and *Trem2*^*−/−*^, we performed pathway analysis using Ingenuity Pathway Analysis (IPA) and Enrichr. As expected, microglial pathways associated with *Trem2* including phagocytosis and Tyrob network were among the most affected pathways (Additional file 4: Figure S1F). RhoA signaling was commonly altered in *Trem2*^Y38C/Y38C^ and *Trem2*^−/−^ mice (Fig. 2d). Rho kinases are involved in pathways regulating cell shape, motility, cell survival and apoptosis. RhoA inhibits oligodendrocyte process extension thus regulating myelination [34]. RhoA/Rho (ROCK) signaling has also been implicated in dendritic spine morphology and axonal growth [35]. Abnormal upregulation of RhoA in *Trem2*^Y38C/Y38C^ and *Trem2*^−/−^ mice, thus, suggests impairments in myelination and synapses.

To further understand the gene expression changes, we performed IPA upstream regulator analysis. Based on the differentially expressed gene, this analysis predicts Upstream regulators that could be activated or inhibited resulting in the observed changes in gene expression. Our analysis indicated a predicted activation of TGFβ-1 upstream regulator in *Trem2*^−/−^ versus WT mice (Fig. 2e). TGFβ-1 signaling has shown to be neuroprotective and increases after injury or stroke [36–38]. Additionally, we found MEF2c (Fig. 2e), a transcription factor that regulates synaptic remodeling in brains of adult mice [39–41], was predicted to be activated in both *Trem2*^Y38C/Y38C^ and *Trem2*^−/−^ mice. Together, these data along with the enrichment analysis provided support that *Trem2* Y38C variant and *Trem2* deficiency induce neuronal dysfunction at 6 months of age.

### *Trem*2^Y38C/Y38C^ and *Trem2*^−/−^ adult mice display reduced myelination

**Fig. 3:**
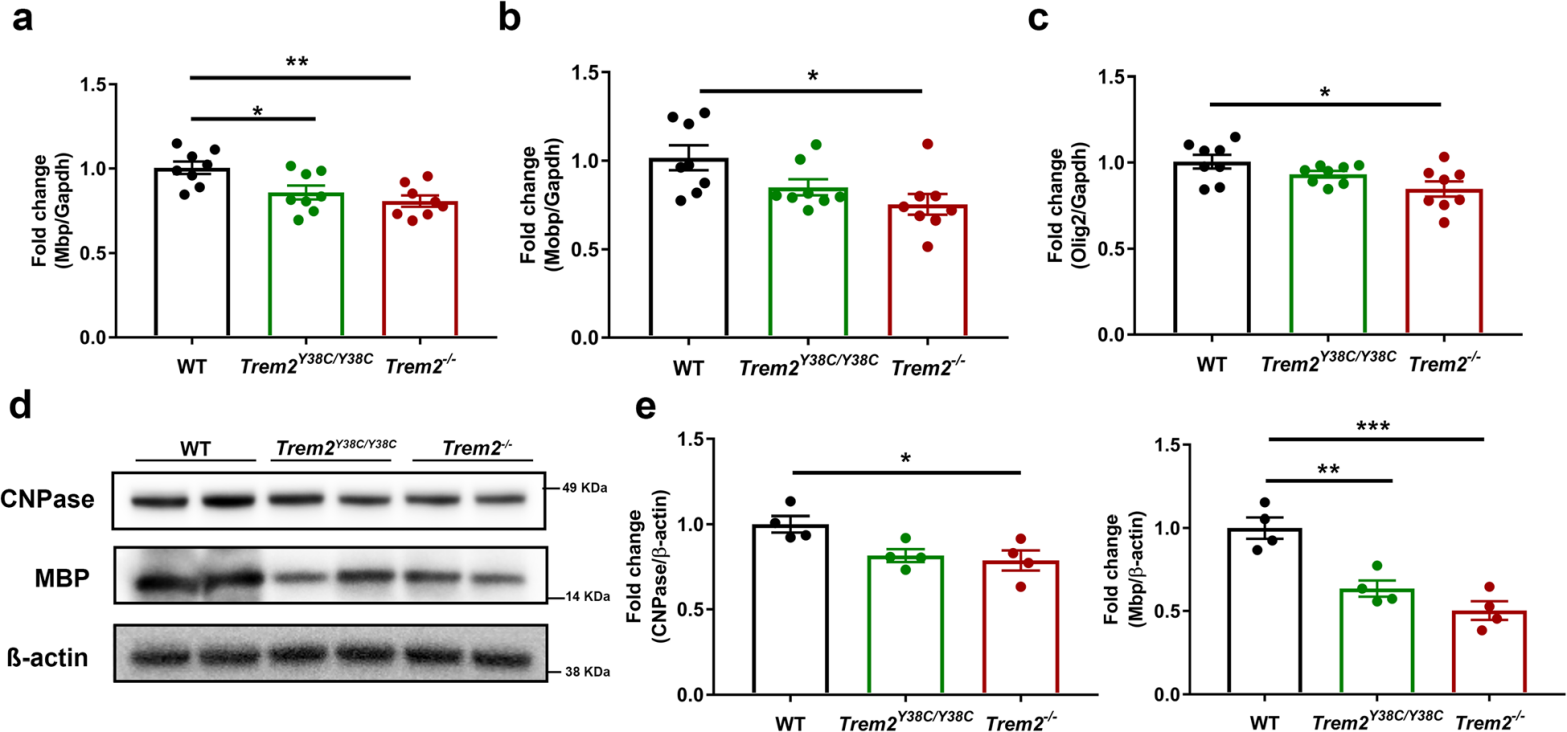
Oligodendrocyte/Myelin impairments in adult *Trem2*^*Y38C/Y38C*^ and *Trem2*^*−/−*^ mice. **a-c** *Mbp, Mobp* and *Olig2* transcript levels were determined by quantitative-PCR. **a** *Mbp* transcripts were reduced in both *Trem2*^*Y38C/Y38C*^ and *Trem2*^*−/−*^ cortical lysates and **b-c** *Mobp* and *Olig2* mRNA reduced significantly in *Trem2*^*−/−*^ cortical lysates. **d** Western blot analysis and **e** Quantification of myelin proteins CNPase and MBP. CNPase levels were reduced significantly in *Trem2*^*−/−*^ mice and Mbp levels were significantly reduced in both *Trem2*^*Y38C/Y38C*^ and *Trem2*^*−/−*^ mice. **a-e** Data represented as mean ± SEM. *N* = 4 (2 males, 2 females); One-way ANOVA with Tukey’s post hoc test; *P < 0.05, **P < 0.01, ***P < 0.001

To further study the oligodendrocyte/myelin alterations observed in the transcriptomics analysis we examined three oligodendrocyte/myelin associated genes, that were downregulated in both *Trem2*^Y38C/Y38C^ and *Trem2*^−/−^, by qPCR. *Myelin basic protein (Mbp)* transcripts were significantly reduction in both *Trem2*^Y38C/Y38C^ and *Trem2*^−/−^ as compared to WT mice (Fig. 3a). *Myelin Associated Oligodendrocyte Basic Protein (Mobp)* and *Oligodendrocyte Transcription Factor 2 (Olig2)* transcripts decreased significantly in *Trem2*^−/−^ mice as compared to the WT mice (Fig. 3b-c). We further studied oligodendrocyte/myelin pathology by analyzing protein expression of CNPase (2′,3′-Cyclic-nucleotide 3′-phosphodiesterase), an enzyme expressed in myelin associated oligodendrocytes, and MBP in cortical lysates of 6 months old WT, *Trem2*^Y38C/Y38C^ and *Trem2*^−/−^ mice. CNPase levels were reduced significantly in *Trem2*^−/−^ as compared to the WT (Fig. 3d-e). MBP protein levels were lowered in both *Trem2*^Y38C/Y38C^ and *Trem2*^−/−^ as compared to WT mice (Fig. 3d-e). Overall, this suggests the presence of altered myelination in *Trem2*^Y38C/Y38C^ and *Trem2*^−/−^ brains.

### Synaptic elements and synaptic plasticity are impaired in *Trem2*^Y38C/Y38C^ and *Trem2*^−/−^ young adults

**Fig. 4:**
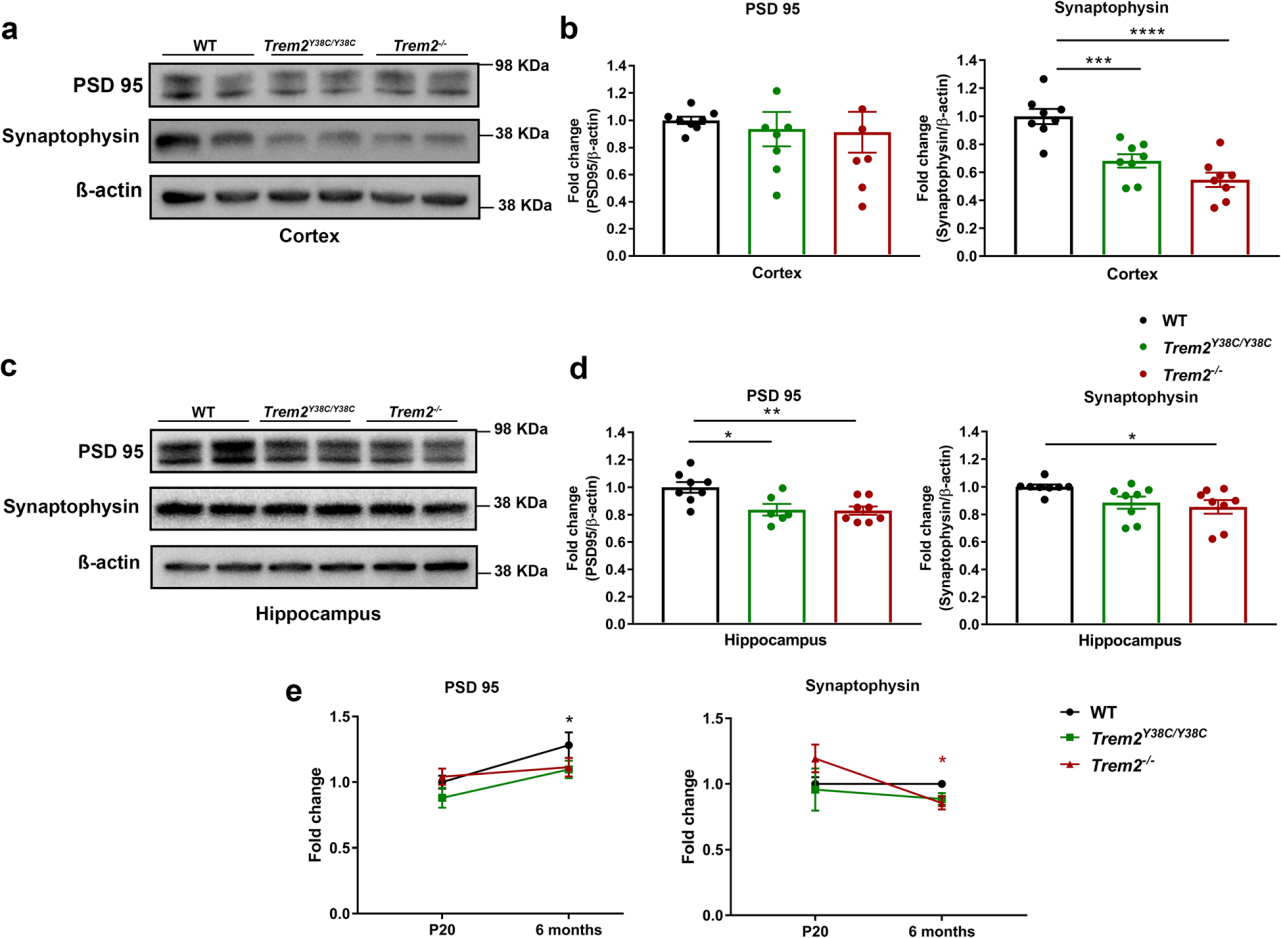
Decreased synaptic elements in adult *Trem2*^*Y38C/Y38C*^ and *Trem2*^*−/−*^ mice. Microdissected **a,b** cortices and **c,d** hippocampi of WT, *Trem2*^*Y38C/Y38C*^ and *Trem2*^*−/−*^ mice were analyzed using western blot with antibodies against PSD95 and synaptophysin. **a,b** *Trem2*^*Y38C/Y38C*^ and *Trem2*^*−/−*^ mice showed significant reduction in synaptophysin but not in PSD-95 protein levels. **c,d** PSD95 and synaptophysin protein levels were significantly reduced in hippocampi of *Trem2*^*Y38C/Y38C*^ and *Trem2*^*−/−*^ mice. **e** Comparison of PSD95 and synaptophysin protein expression in hippocampal lysates of WT, *Trem2*^*Y38C/Y38C*^ and *Trem2*^*−/−*^ mice at P20 and 6 months. Comparing P20 to 6 months, PSD95 levels increase significantly (black asterisk) in WT mice (black), while both *Trem2*^*Y38C/Y38C*^ (green) and *Trem2*^*−/−*^ (red) mice show comparatively less increase resulting in reduced PSD95 levels at 6 months as compared to WT. Synaptophysin levels were maintained in WT mice (black) and *Trem2*^*Y38C/Y38C*^ (green), while *Trem2*^*−/−*^ (red) mice showed significantly lower levels at 6 months as compared to P20 (red asterisk) indicating loss of presynaptic elements in adult mice. Sample sizes for each group: *N* = 6–8 (3–4 each, males and females). Data are shown as mean ± SEM. **b,d** One-way ANOVA with Tukey’s post hoc test; *P < 0.05, **P < 0.01, ***P < 0.001, **** *P* < 0.0001. **e** Two-way ANOVA with Bonferroni’s post hoc test; *P < 0.05, WT mice at 6 months versus P20 (black) and *Trem2*^*−/−*^ mice at 6 months versus P20 (red)

Transcriptomics analysis also suggested alteration of genes associated with neuronal function. To validate and extend these finding in 6 months old *Trem2*^Y38C/Y38C^ and *Trem2*^−/−^ mice, we first evaluated synaptic protein levels in cortex and hippocampus using western blot. In the cortex, no changes were observed in postsynaptic protein PSD-95 levels but presynaptic protein synaptophysin levels were significantly reduced in both *Trem2*^Y38C/Y38C^ and *Trem2*^−/−^ mice (Fig. 4a,b). To confirm that the differences in presynaptic and postsynaptic proteins were not specific to these markers, we examined additional synaptic proteins. We observed no changes in Homer1 levels but noted significant decreases in synapsin 2 levels in cortices of *Trem2*^−/−^ mice (Additional file 11: Figure S3A-B). The hippocampal protein lysates showed decreased pre- and postsynaptic elements with reduction in both PSD-95 and synaptophysin levels in *Trem2*^Y38C/Y38C^ and *Trem2*^−/−^ mice (Fig. 4c,d). Thus, region-specific changes in synaptic proteins were observed at 6 months with more alterations in hippocampal synapses.

To investigate if these alterations were initiated during development or were induced post-development, we evaluated synaptic elements in cortical and hippocampal lysates of WT, *Trem2*^Y38C/Y38C^, and *Trem2*^−/−^ mice at P20. Neither group demonstrated alterations in synaptic protein levels at P20 (Additional file 11: Figure S3C-D). Additionally, we compared pre- and postsynaptic proteins in the hippocampi of P20 mice to that of 6 months old mice. PSD95 levels increased significantly with age in WT mice; while *Trem2*^Y38C/Y38C^ and *Trem2*^−/−^ mice didn’t display similar increase over the time, which resulted in reduced PSD95 levels in *Trem2*^Y38C/Y38C^ and *Trem2*^−/−^ as compared to WT mice at 6 months of age (Fig. 4e). This suggests that the decrease in PSD95 could be due to altered development of post-synaptic elements in adult *Trem2*^Y38C/Y38C^ and *Trem2*^−/−^ mice. Synaptophysin levels were maintained in WT hippocampi from P20 to 6 months of age whereas, 6 months old *Trem2*^−/−^ mice displayed synaptophysin levels lower than their respective P20 levels. This suggests that absence of *Trem2* might induce active loss of pre-synaptic elements in adult mice. Thus, this decreased abundance of synaptic proteins in *Trem2*^Y38C/Y38C^ and *Trem2*^−/−^ mice at 6 months may stem from a combination of altered synaptic development and loss of synaptic elements in adult mice. Next, we determined if the severe hippocampal alterations in synaptic elements correlated with altered microglial abundance and Iba1+ hippocampal area. Iba-1+ cell counts did not change significantly (Fig Additional file 11: Figure S3E), however, percentage of area occupied by Iba-1+ cells significantly increased in both *Trem2*^Y38C/Y38C^ and *Trem2*^−/−^ hippocampus (Fig Additional file 11: Figure S3F).

**Fig. 5:**
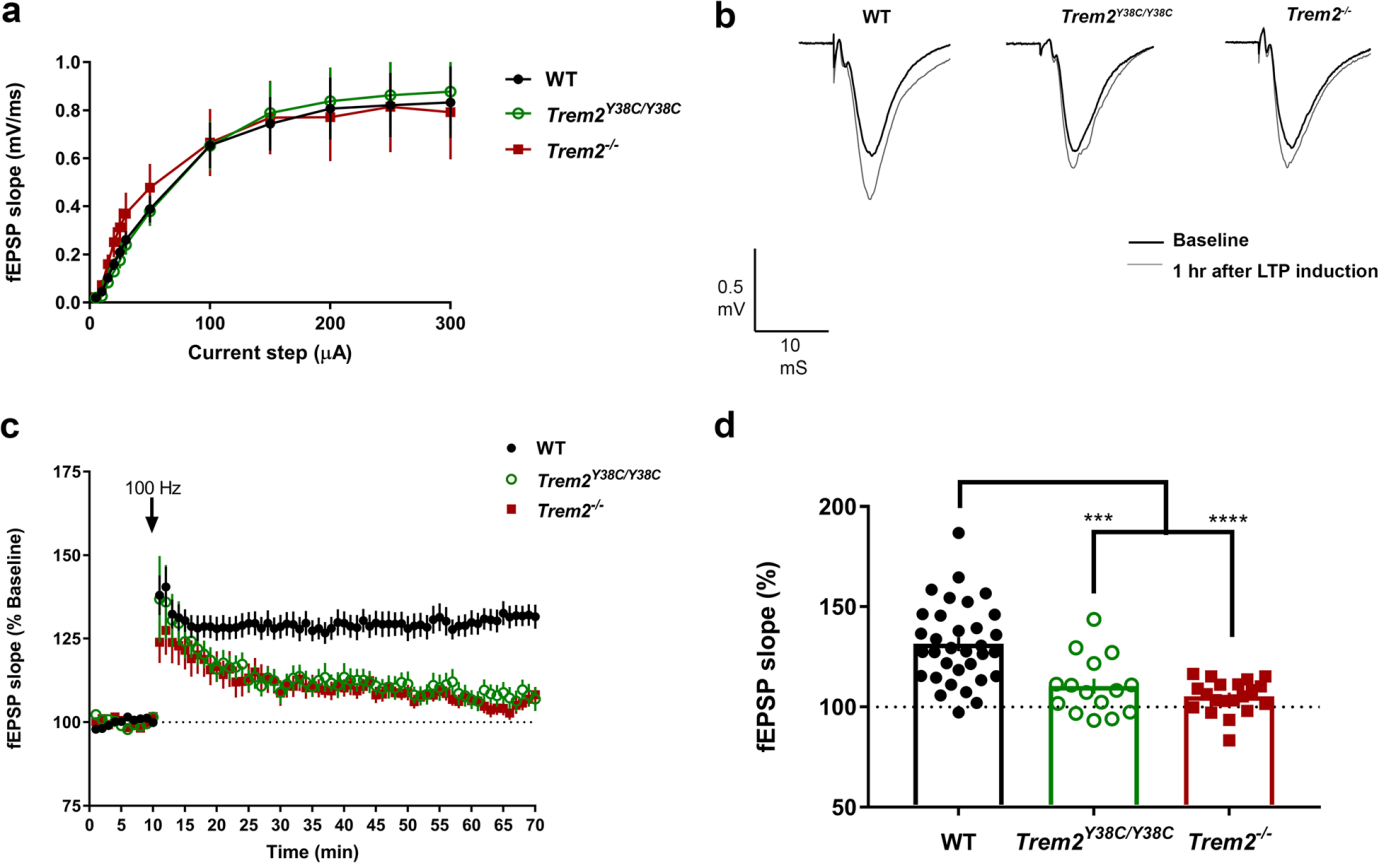
Hippocampal synaptic plasticity is reduced in *Trem2*^*Y38C/Y38C*^ and *Trem2*^*−/−*^ mice. **a** Input-output curves were similar in *Trem2*^*Y38C/Y38C*^, *Trem2*^*−/−*^, and WT mice. **b,c** Hippocampal Schaffer collateral-CA1 LTP is significantly reduced in *Trem2*^*Y38C/Y38C*^ (green) and *Trem2*^*−/−*^ (red) mice as compared to WT mice (black) at 6 months of age. **b** Superimposed representative traces before (black) and 60 min after high-frequency stimulation (gray) in each genotype. **c** Mean time course of fEPSP slope in hippocampal slices from WT (black), *Trem2*^*Y38C/Y38C*^ (green) and *Trem2*^*−/−*^ (red). Arrow indicates time of high frequency stimulation. **d** Average of normalized fEPSP slope for final 10 min of recording (60–70 min) relative to 10-min baseline average (dotted line). Data are shown as mean ± SEM. Sample sizes: WT mice, *N* = 15 (7 females and 8 males, *n* = 33 recordings); *Trem2*^*Y38C/Y38C*^ mice, *N* = 7 (3 females and 4 males, *n* = 20 recordings); *Trem2*^*−/−*^ mice, N = 8 (4 males and 4 females, *n* = 15 recordings). One-way ANOVA with Tukey’s post hoc test. ***P < 0.001, **** *P* < 0.0001

Lastly, to evaluate the impact of alterations of hippocampal synaptic protein on synaptic functions, we assessed induction of long-term potentiation (LTP) in hippocampal area CA1 by stimulating Schaffer collaterals in brain slices of 6 months old WT, *Trem2*^Y38C/Y38C^ and *Trem2*^−/−^ mice. No differences were detected in input/output curves or paired-pulse facilitation (Fig. 5a, Additional file 8: Figure S4). However, a statistically significant reduction in the magnitude of LTP was observed in *Trem2*^Y38C/Y38C^ and *Trem2*^−/−^ LTP responses as compared to WT (Fig. 5b-d). These results indicate severe impairment in hippocampal neuronal plasticity in *Trem2*^Y38C/Y38C^ and *Trem2*^−/−^ mice at 6 months.

## Discussion

A common clinical manifestation in NHD patients and some of the genetic variants of TREM2 or TYROBP is early-onset dementia. To understand how loss of functional TREM2 confers predisposition to early-onset dementia, we used *Trem2*^*Y38C/Y38C*^ and *Trem2*^*−/−*^ mouse models to study neuronal pathology at a developmental and 6 months age. Importantly, as observed in the brains of humans harboring *TREM2* variants [42], the endogenous expression levels of TREM2 Y38C transcript levels were comparable to the WT. This suggests there were no aberrant alterations in *Trem2* mRNA, which was reported in *Trem2* R47H mouse model [21, 29]. Similarly, no alterations were observed in protein expression of TREM2 and sTREM2 in *Trem2*^*Y38C/Y38C*^ mice. Further studies are required to determine the proportion of TREM2 receptor expression at the plasma membrane retention to its retention in endoplasmic reticulum in vivo.

Since TREM2 is expressed exclusively on microglia in the brain, we analyzed microglia number and morphology in the cortices of *Trem2*^*Y38C/Y38C*^ and *Trem2*^*−/−*^ mice and found no significant changes in the number of Iba-1 positive microglia. However, we found increased area covered by Iba-1 positive microglia with increased number of branches which is in support of the previous findings [19, 20]. The increased microglial ramification of microglial processes suggests in *Trem2*^*Y38C/Y38C*^ and *Trem2*^*−/−*^ mice as compared to WT microglia is reminiscent of the “homeostatically locked” state that has been described for Trem2 deficient microglia [43]. Studying *Trem2*^*Y38C/Y38C*^ and *Trem2*^*−/−*^ microglia in presence of a pathological trigger such as amyloid can provide further information on activation capacity of microglia with mutant TREM2 or lack of TREM2.

Another manifestation in NHD patients with TREM2 dysfunction is development of FTD-like dementia with distinct white matter loss [10, 44]. Weighted gene co-expression network analysis of Velocigene *Trem2*^−/−^ mice also showed oligodendrocyte/myelin related modules as one of the most disrupted cell type modules [45]. Likewise, *Trem2*^*Y38C/Y38C*^ and *Trem2*^*−/−*^ mice revealed alterations in oligodendrocytes/myelination based on the transcriptomics analysis. Since this suggested white matter dysfunction, we expanded our analysis of oligodendrocyte specific genes in *Trem2*^Y38C/Y38C^ and *Trem2*^−/−^ mice brains. Among the many downregulated oligodendrocyte/myelin genes, Mbp (myelin basic protein) and Mobp (myelin associated oligodendrocyte basic protein) were downregulated in both *Trem2*^*Y38C/Y38C*^ and *Trem2*^*−/−*^. Expression of myelin proteins such as MBP and CNPase were reduced in *Trem2*^*Y38C/Y38C*^ and *Trem2*^*−/−*^. IPA analysis revealed ILK signaling, which is involved in oligodendrocyte development [46], was impacted in both *Trem2*^Y38C/Y38C^ and *Trem2*^−/−^ mice. We observed significant reduction in MBP protein expression in the cortical lysates of *Trem2*^Y38C/Y38C^ and *Trem2*^−/−^ mice, further supporting our transcriptomics findings. Our observed oligodendrocytes/myelin alterations in cortices of the *Trem2*^Y38C/Y38C^ and *Trem2*^−/−^ young adult mice suggest nascent white-matter alterations in these mice. This is concordant with the reports highlighting the role of microglia and TREM2 in white matter physiology. Further detailed studies for analyzing white-matter tracts, either by brain scans or electron microscopy, in older mice are necessary to determine the severity and progression of the pathology.

Microglia support myelination by taking up myelin debris and stimulating oligodendrocyte maturation after demyelinating events [47, 48]. TREM2 contributes to this process by sensing lipids and phagocytosing myelin debris. In the absence of TREM2 in mouse models of demyelination, microglia take up myelin debris but fail to degrade it, resulting in the accumulation of lipids and persistent demyelination [49, 50]. With aging, *Trem2* T66M mice exhibit reduced microglial activity and microglial clusters in white matter tracts [11]. Similarly, our findings highlight the role of microglial TREM2 in oligodendrocytes/myelin maintenance. Recently, using single-nuclei transcriptomics of human samples, AD-reactive oligodendrocytes were shown to increase significantly in TREM2-R62H carries as compared to the controls but seem to be lower than TREM2-common variant samples [51]. The study also reported downregulation of genes in Oligo1 cluster (includes genes such as Mbp, Mobp and Olig2) in early-onset AD which corresponds to our findings that indicate reduction in oligodendrocyte/myelin markers in *Trem2*^Y38C/Y38C^ and *Trem2*^−/−^ young adult mice.

We found perturbations in gene expression associated with neuronal functions in the cortex of adult *Trem2*^Y38C/Y38C^ and *Trem2*^−/−^ mice. IPA upstream regulator analysis was suggestive of synaptic remodeling as it predicted activation of upstream transcription factor Mef2c (Myocyte-specific enhancer factor 2C). Mef2c is regulated by synaptic activity and important in neuronal migration, synapse development and synaptic transmission [52]. Presynaptic proteins such as synaptophysin and synapsin, were reduced in the cortex and hippocampus, while levels of postsynaptic proteins, PSD95 and Homer1, were reduced only in the hippocampus. This suggests enhanced synapse-associated pathology in hippocampus of *Trem2*^Y38C/Y38C^ and *Trem2*^−/−^ mice at 6 months. These differences in regional specificity could be due to regional diversity observed in glial cells [53, 54]. To understand if the reduced synaptic protein levels were reflective of a developmental defect or a post-developmental phenomenon, we analyzed the synaptic markers in cortex and hippocampus at P20 and 6 months age. Previous reports have observed increased synaptic proteins in the hippocampus of *Trem2*^−/−^ mice at P20 [19] or no changes were reported in 1 month old *Trem2*^*−/−*^ mice [20]. Our data demonstrate no changes in synaptic protein expression at P20 in *Trem2*^*Y38C/Y38C*^ and *Trem2*^*−/−*^ mice. These results indicate that synaptic protein levels are not affected during development but decrease in adult mice. Analysis of synaptic proteins across P20 and 6 months ages suggested decreased abundance of synaptic proteins in *Trem2*^Y38C/Y38C^ and *Trem2*^−/−^ mice might be a combination of altered synaptic development and active loss of synaptic elements in adult mice. These synaptic alterations further influenced deficits in hippocampal synaptic plasticity as observed by significant reduction in hippocampal LTP. LTP at the Schaffer collateral-CA1 synapse is thought to be crucial for the cognitive process involved in learning and memory. Spatial memory deficits have also been reported in 12 months old *Trem2*^−/−^ mice [55]. *Trem2*^Y38C/Y38C^ and *Trem2*^−/−^ adult mice exhibited abnormal behaviors such as excess self-grooming or barbering leading to hairless patches (data not shown and as reported previously [19]). However, more extensive battery of behavioral tests is needed to characterize impairments in behavior.

Pathway enrichment analysis revealed alterations in RhoA signaling in *Trem2*^*Y38C/Y38C*^ and *Trem2*^*−/−*^ mice. Rho signaling is involved in cytoskeleton remodeling and inhibition of RhoA triggers axonal growth cone [56]. RhoA inhibitors not only increase myelination in injury models but also restore synaptic plasticity in an anxiety mouse model [57, 58]. Altered Rho signaling and microglial activation could potentially provide molecular insights on underlying process the oligodendrocyte/myelin and synaptic dysfunction in *Trem2*^*Y38C/Y38C*^ and *Trem2*^*−/−*^ mice. However, further investigation is needed to understand if this pathway is altered in a specific or multiple cell type.

As previously reported [19, 20], our data indicate TREM2 is required to maintain proper synaptic balance. We expand these findings to demonstrate that loss of functional TREM2 negatively impacts neuronal synapses and oligodendrocytes/myelin post-developmentally. Interestingly, mice deficient in DAP12, a signaling adapter of various immunoreceptors including TREM2, also show hypomyelination and synaptic degeneration in thalamus as early as 3 months [59]. As dysmyelination has shown to alter synaptic transmission and in turn impact synapse integrity [60–62], we suspect that the alteration in synaptic integrity and transmission in absence of TREM2 could be a secondary effect of oligodendrocyte/myelin impairment due to reduced microglial activity. RhoA could potentially be the pathway orchestrating this pathology. *Trem2*^Y38C/Y38C^ mice demonstrated pathological changes that were similar to *Trem2*^−/−^ mice including several overlapping differentially expressed genes and significant changes in microglial morphology and synaptic protein levels. Functional outcomes were analogous since the reduction in LTP responses were comparable *Trem2*^*Y38C/Y38C*^ and *Trem2*^*−/−*^ mice. Recently, TREM2-R47H mutation has also shown to reduce LTP in young rats [63]. In normal human brains, *Trem2* expression is higher in white matter and hippocampus as compared to other brain regions [64]. Interestingly, our findings display *Trem2*^Y38C/Y38C^ and *Trem2*^−/−^ mice have reduced myelination and synaptic alterations are most prominent in the hippocampus. Overall, *Trem2*^Y38C/Y38C^ and *Trem2*^−/−^ young adult mice present phenotypic characteristics reminiscent of the clinical manifestations in patients with NHD. The synaptic dysfunction along with oligodendrocyte/myelin impairment observed at 6 months in *Trem2*^Y38C/Y38C^ and *Trem2*^−/−^ mice could help explain why *Trem2* variants are predisposed to early onset dementia. Collectively, the continuation of studies that dissect the mechanisms by which loss of functional TREM2 in microglia directly and indirectly, through microglial modulation of other cell types, impact synaptic functions and white matter could unveil mechanistic role of TREM2 in brain homeostasis and disease.

## Conclusion

In summary, our findings contribute in vivo evidence that TREM2 Y38C disrupts normal TREM2 functions. *Trem2*^Y38C/Y38C^ and *Trem2*^−/−^ mice demonstrated altered gene expression, changes in microglia morphology, loss of synaptic proteins, reduced oligodendrocyte/myelin specific transcripts and impaired hippocampal synaptic plasticity. These results explain some of the early events leading to presenile dementia. Loss of TREM2 signaling negatively impacts neuronal function post-developmentally and offers insight to how TREM2 alterations can confer synaptic impairment. Future studies are needed to determine if these events precede neuronal susceptibility to pathological triggers such as amyloid.

## Supplementary information

**Additional file 1: Table S1.** Primers for amplification of genomic regions predicted to have off-target mutations from CRISPR/Cas9.

**Additional file 2: Table S2.** Primers for Sanger’s sequencing of predicted off-target mutations from CRISPR/Cas9.

**Additional file 3: Table S3.** *Trem2*^Y38C/Y38C^ mice do not display aberrant expression of genes upstream or downstream of *Trem2.* Log fold change (logFC) with their FDRs are shown for genes within 100 kb upstream and downstream of *Trem2*. Results are normalized to gene expression in WT mice. N/A = not available.

**Additional file 4: Figure S1.** (A,B) Heatmap representations for differentially expressed genes in (A) *Trem2*^*Y38C/Y38C*^ versus WT mice and (B) *Trem2*^*−/−*^ versus WT mice. (C) Venn diagram showing the overlap of significantly upregulated (blue) and downregulated (gray) differentially expressed genes between *Trem2*^*Y38C/Y38C*^ and *Trem2*^*−/−*^ mice compared to wildtype mice . (D-G) Using Enrichr web- tools, downregulated genes shared between *Trem2*^*Y38C/Y38C*^ and *Trem2*^*−/−*^ mice were analyzed for tissue enrichment (D) enrichment of genes highlighting association of downregulated genes with cortical layers. Cellular compartment enrichment includes (E) myelin sheath, (F) Pathway enrichment showing alterations in microglial phagocytosis-associated pathway. (D-F) *P* values are indicated at the end of each colored bar. Shaded gets lighter with smaller *P* value. Sample sizes: WT mice, *N* = 8 (4 males, 4 females); *Trem2*^*Y38C/Y38C*^ mice, N = 8 (4 males, 4 females); *Trem2*^*−/−*^ mice, *N* = 5 (1 male, 4 females).

**Additional file 5: Table S4.** CSV file. List of differentially expressed genes in cortices of *Trem2*^*Y38C/Y38C*^ versus WT mice. N=8 (equal number of males and females).

**Additional file 6: Table S5.** CSV file. List of differentially expressed genes in cortices of *Trem2*^*-/*^*-* versus WT mice mice. N=5 (1 male, 4 females).

**Additional file 7: Table S6.** CSV file. List of differentially expressed genes in cortices of *Trem2*^*Y38C/Y38C*^ versus *Trem2*^*-/*^*-* mice. N=5 (1 male, 4 females).

**Additional file 8: Figure S2.** (A) Volcano plot illustrating differentially expressed genes (*P*<0.05) in *Trem2*^*Y38C/Y38C*^ versus *Trem2*^*-/*^*-* cortices. (B-C) Pathway enrichment of downregulated and upregulated genes (FDR<0.05) by Enrichr. (B) Pathway enrichment for downregulated genes indicates alteration in circadian rhythm associated pathways in *Trem2*^*Y38C/Y38C*^ as compared to *Trem2*^*-/*^*-* mice (C) Upregulated genes show enrichment in pathways associated with protein processing in endoplasmic reticulum in *Trem2*^*Y38C/Y38C*^ versus *Trem2*^*-/-*^. (B-C) P values are indicated at the end of each colored bar. Shaded gets lighter with smaller P value. Red bars represent enrichment for downregulated genes and green bars represent enrichment for upregulated genes. Sample sizes: WT mice, *N* = 8 (4 males, 4 females); Trem2Y38C/Y38C mice, N = 8 (4 males, 4 females); Trem2-/- mice, N = 5 (1 male, 4 females).

**Additional file 9: Table S7.** Downregulated genes associated with oligodendrocyte/myelin in *Trem2*^Y38C/Y38C^ versus WT mice. logFC = Log fold change.

**Additional file 10: Table S8.** Downregulated genes associated with oligodendrocyte/myelin genes *Trem2*^*-/*^*-* versus WT mice. logFC = Log fold change.

**Additional file 11: Figure S3.** (A,B) Western blot analysis was performed on cortical lysates of 6 months old WT, *Trem2*^Y38C/Y38C^ and *Trem2*^-/^- mice. (B) Quantification of western blot bands shows decreased synapsin 2 levels in *Trem2*^-/^- mice with no changes in Homer1 levels. (C,D) Western blots and quantification of PSD95 and synaptophysin in (C) cortical and (D) hippocampal lysates of P20 WT, *Trem2*^Y38C/Y38C^ and *Trem2*^-/^- mice. (E) Iba-1+ cell count in the hippocampus of 6 months WT, *Trem2*^Y38C/Y38C^ and *Trem2*^-/^- mice showed no significant change (F) Iba-1 percent area in the hippocampus increased significantly in both *Trem2*^Y38C/Y38C^ and *Trem2*^-/^- as compared to the WT. (B-D) Data represented as mean ± SEM. Sample size: N = 8 (4 males and 4 females). One-way ANOVA with Tukey’s post hoc test. *** *p*< 0.001. (E-F).

**Additional file 12: Figure S4.** WT, *Trem2*^Y38C/Y38C^ and *Trem2*^−/−^ mice exhibit similar paired-pulse ratio (PPR). Paired-pulse ratio is the amplitude of excitatory postsynaptic current (EPSC) 2/EPSC1. ISI = interstimulus interval. Data are shown as mean ± SEM. Sample size: WT mice, *N* = 15 (7 females and 8 males), *n* = 33 recordings; *Trem2*^*Y38C/Y38C*^ mice, *N* = 7 (4 males and 3 females), *n* = 20 recordings; *Trem2*^*−/−*^ mice, *N* = 8 (4 males and 4 females), *n* = 15 recordings.

## Data Availability

The datasets used and/or analyzed during the current study are provided as additional files and are available from the corresponding author on reasonable request.

## Abbreviations

NHD: Nasu Hakola Disease
FTD: Fronto-Temporal Dementia
AD: Alzheimer’s disease
CRISPR: Clustered regularly interspaced short palindromic repeats
PAM: Protospacer adjacent motif
*Trem2*: Triggering Receptor expressed on myeloid cells 2
Iba-1: Ionized calcium-binding adapter molecule 1
RhoA: Ras homolog family member A
P20: Postnatal day 20
Tyrobp: TYRO protein tyrosine kinase binding protein
DAP12: DNAX-Activation Protein 12
LTP: Long term potentiation
PSD95: Postsynaptic density protein 95
Mef2c: Myocyte Enhancer Factor 2C
IPA: Ingenuity pathway analysis
