# Supplementary material for: Trem2 Y38C mutation and loss of Trem2 impairs neuronal synapses in adult mice
Source: Mol Neurodegener. 2020 Oct 28;15:62. doi: 10.1186/s13024-020-00409-0 (PMC7594478; doi:10.1186/s13024-020-00409-0)
Supplement: Supplementary file 1 — Additional file 1: Table S1. Primers for amplification of genomic regions predicted to have off-target mutations from CRISPR/Cas9. [file 13024_2020_409_MOESM1_ESM.docx]

**Additional file 1:**

**Table S1. Primers for amplification of genomic regions predicted to have off-target mutations from CRISPR/Cas9.**

| Site 1 | Forward: 5’ GGGTGAGACCAACAGGATGG 3’  Reverse: 5’ CTTCCCCCTTGCTCTATGGT 3’ |
| --- | --- |
| Site 2 | Forward: 5’ TCTGTGTTTAAGCCCCTCCTC 3’  Reverse: 5’ TGATCAGAAGAGCCAGCACA 3’ |
| Site 3 | Forward: 5’ ACTCAAACCACCTGCTTGGG 3’  Reverse: 5’ GGAATTAGGACCTGAGAGGGC 3’ |
| Site 4 | Forward: 5’ ATGAGAACCTAAGTACCAAGTGCT 3’  Reverse: 5’ CTTCTTCTTATCTTCTTGATGCTACA 3’ |
