# Supplementary material for: Trem2 Y38C mutation and loss of Trem2 impairs neuronal synapses in adult mice
Source: Mol Neurodegener. 2020 Oct 28;15:62. doi: 10.1186/s13024-020-00409-0 (PMC7594478; doi:10.1186/s13024-020-00409-0)
Supplement: Supplementary file 2 — Additional file 2: Table S2. Primers for Sanger’s sequencing of predicted off-target mutations from CRISPR/Cas9. [file 13024_2020_409_MOESM2_ESM.docx]

**Additional file 2:**

**Table S2. Primers for Sanger’s sequencing of predicted off-target mutations from CRISPR/Cas9.**

| Site 1 | 5’ GCTACAGAGAAATCCCATGATAGA 3’ |
| --- | --- |
| Site 2 | 5’ GGTCTTATCTGCACACCTATACA 3’ |
| Site 3 | 5’ AGATAGGGTGACTCAGCTCTGTAC 3’ |
| Site 4 | 5’ GTCTCTACACAGCACAGTCTCTC 3’ |
