# Supplementary material for: Trem2 Y38C mutation and loss of Trem2 impairs neuronal synapses in adult mice
Source: Mol Neurodegener. 2020 Oct 28;15:62. doi: 10.1186/s13024-020-00409-0 (PMC7594478; doi:10.1186/s13024-020-00409-0)
Supplement: Supplementary file 3 — Additional file 3: Table S3. Trem2Y38C/Y38C mice do not display aberrant expression of genes upstream or downstream of Trem2. Log fold change (logFC) with their FDRs are shown for genes within 100 kb upstream and downstream of Trem2. Results are normalized to gene expression in WT mice. N/A = not available. [file 13024_2020_409_MOESM3_ESM.docx]

**Additional file 3:**

**Table S3**. *Trem2*^Y38C/Y38C^ mice do not display aberrant expression of genes upstream or downstream of *Trem2.* Log fold change (logFC) with their FDRs are shown for genes within 100 kb upstream and downstream of *Trem2*. Results are normalized to gene expression in WT mice. N/A = not available.

| **Genes 100Kb ±** | ***Trem2^Y38C/Y38C^*** | | ***Trem2^-/-^*** | |
| --- | --- | --- | --- | --- |
|  | **logFC** | **adjusted P-value** | **logFC** | **adjusted P-value** |
| B430306N03Rik | -0.193405748 | N/A | -0.509583346 | N/A |
| Apobec2 | N/A | N/A | 29.92457187 | 0.061861301 |
| Tspo2 | 1.258532791 | 0.683681926 | 0.570343668 | 0.631277204 |
| Oard1 | 0.931680738 | 0.848378799 | 1.683472756 | 0.25996477 |
| Nfya | 1.019403231 | 0.892929577 | 1.516241707 | 0.353580637 |
| Treml2 | 0.528146326 | 0.278614266 | 0.700364131 | 0.729182964 |
| Treml1 | 1.053512506 | N/A | 1.094334288 | N/A |
