# Supplementary figures and images for: Trem2 Y38C mutation and loss of Trem2 impairs neuronal synapses in adult mice
Source: Mol Neurodegener. 2020 Oct 28;15:62. doi: 10.1186/s13024-020-00409-0 (PMC7594478; doi:10.1186/s13024-020-00409-0)

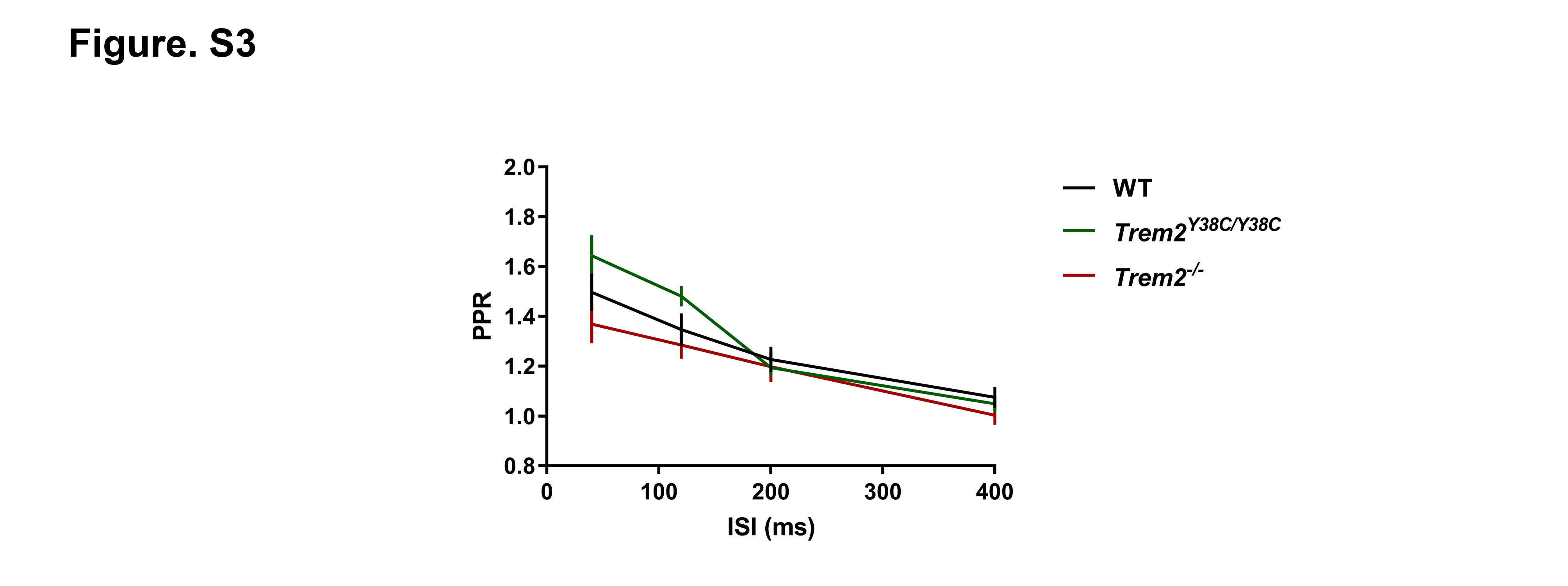

Supplement: Supplementary file 8 — Additional file 8: Figure S2. (A) Volcano plot illustrating differentially expressed genes (P<0.05) in Trem2Y38C/Y38C versus Trem2-/- cortices. (B-C) Pathway enrichment of downregulated and upregulated genes (FDR<0.05) by Enrichr. (B) Pathway enrichment for downregulated genes indicates alteration in circadian rhythm associated pathways in Trem2Y38C/Y38C as compared to Trem2-/- mice (C) Upregulated genes show enrichment in pathways associated with protein processing in endoplasmic reticulum in Trem2Y38C/Y38C versus Trem2-/-. (B-C) P values are indicated at the end of each colored bar. Shaded gets lighter with smaller P value. Red bars represent enrichment for downregulated genes and green bars represent enrichment for upregulated genes. Sample sizes: WT mice, N = 8 (4 males, 4 females); Trem2Y38C/Y38C mice, N = 8 (4 males, 4 females); Trem2-/- mice, N = 5 (1 male, 4 females). [file 13024_2020_409_MOESM8_ESM.zip › Additional file 8.tif]

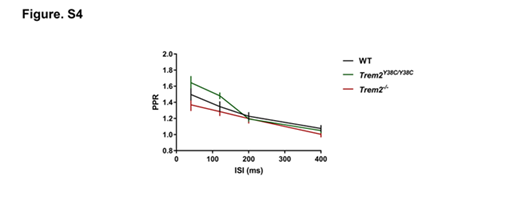

Supplement: Supplementary file 12 — Additional file 12: Figure S4. WT, Trem2Y38C/Y38C and Trem2−/− mice exhibit similar paired-pulse ratio (PPR). Paired-pulse ratio is the amplitude of excitatory postsynaptic current (EPSC) 2/EPSC1. ISI = interstimulus interval. Data are shown as mean ± SEM. Sample size: WT mice, N = 15 (7 females and 8 males), n = 33 recordings; Trem2Y38C/Y38C mice, N = 7 (4 males and 3 females), n = 20 recordings; Trem2−/− mice, N = 8 (4 males and 4 females), n = 15 recordings. [file 13024_2020_409_MOESM12_ESM.png]
