# Supplementary material for: Trem2 Y38C mutation and loss of Trem2 impairs neuronal synapses in adult mice
Source: Mol Neurodegener. 2020 Oct 28;15:62. doi: 10.1186/s13024-020-00409-0 (PMC7594478; doi:10.1186/s13024-020-00409-0)
Supplement: Supplementary file 9 — Additional file 9: Table S7. Downregulated genes associated with oligodendrocyte/myelin in Trem2Y38C/Y38C versus WT mice. logFC = Log fold change. [file 13024_2020_409_MOESM9_ESM.docx]

**Additional file 9:**

**Table S7:** Downregulated genes associated with oligodendrocyte/myelin in *Trem2*^Y38C/Y38C^ versus WT mice. logFC = Log fold change.

| **Genes** | **logFC** | **Adjusted P-value** |
| --- | --- | --- |
| Mobp | -0.51489 | 5.63E-09 |
| Mal | -0.5044 | 2.29E-06 |
| Bcas1 | -0.45522 | 0.000217 |
| Mbp | -0.43996 | 5.21E-05 |
| Kif5a | -0.40209 | 6.42E-06 |
| Plp1 | -0.39414 | 0.001243 |
| Tmem88b | -0.38728 | 0.000321 |
| Pllp | -0.36739 | 0.029035 |
| Fa2h | -0.36064 | 0.011426 |
| Plekhb1 | -0.34122 | 0.010829 |
| Olig2 | -0.29125 | 0.019423 |
| Trf | -0.28881 | 0.002642 |
| Prr18 | -0.28773 | 0.014478 |
| Myrf | -0.28694 | 0.01136 |
| Ndrg1 | -0.27351 | 0.004357 |
| Gpr37 | -0.27212 | 0.018013 |
| Mog | -0.23858 | 0.042384 |
| Ugt8a | -0.19711 | 0.019234 |
| Trp53inp2 | -0.15805 | 0.025416 |
