# Supplementary material for: Trem2 Y38C mutation and loss of Trem2 impairs neuronal synapses in adult mice
Source: Mol Neurodegener. 2020 Oct 28;15:62. doi: 10.1186/s13024-020-00409-0 (PMC7594478; doi:10.1186/s13024-020-00409-0)
Supplement: Supplementary file 10 — Additional file 10: Table S8. Downregulated genes associated with oligodendrocyte/myelin genes Trem2-/- versus WT mice. logFC = Log fold change. [file 13024_2020_409_MOESM10_ESM.docx]

**Additional file 10:**

**Table S8:** Downregulated genes associated with oligodendrocyte/myelin genes *Trem2^-/-^* versus WT mice. logFC = Log fold change.

| **Genes** | **logFC** | **Adjusted P-value** |
| --- | --- | --- |
| Olig2 | -0.55751 | 0.002462 |
| Cryab | -0.51833 | 0.006182 |
| Mbp | -0.45016 | 0.001463 |
| Mobp | -0.42102 | 0.004329 |
| Bcas1 | -0.39897 | 0.027634 |
| Ugt8a | -0.29526 | 0.035691 |
| Trp53inp2 | -0.22736 | 0.006418 |
